# Lipid Control and Medical Costs Among Patients With and Without Established Atherosclerotic Cardiovascular Disease Followed in a Brazilian Private Healthcare System

**DOI:** 10.5334/gh.1345

**Published:** 2024-08-14

**Authors:** Pedro Gabriel Melo de Barros e Silva, Henry Szneider, Diego Ribeiro Garcia, Valter Furlan, Renato Delascio Lopes

**Affiliations:** 1 United Health Group Brazil, São Paulo, Brazil; 2 Brazilian Clinical Research Institute, São Paulo, Brazil; 3 Centro Universitário São Camilo, São Paulo, Brazil

**Keywords:** Cardiovascular Diseases, Guideline Adherence, Registries

## Abstract

**Background:**

There is limited real-world data of lipid control and healthcare costs among patients with and without Atherosclerotic Cardiovascular Disease (ASCVD) in Latin America.

**Methods:**

A retrospective cohort study including patients with LDL-cholesterol (LDL-C) assessment from 2015 to 2017 was performed in a health insurance database. Patient characteristics, comorbidities and laboratory data were collected, and International Classification of Diseases (ICD) codes were used to identify a subcohort of patients with ASCVD (secondary prevention) and assess the proportion of these patients with LDL-C controlled. Lipid control among patients without ASCVD (primary prevention) and healthcare costs in one year in the overall population were also assessed.

**Results:**

From the 17,434 patients selected, 5,208 (29.8%) had ASCVD. The mean age of these patients in secondary prevention was 68.9 (±12.3) years and 47.8% were male patients. LDL-C < 70 mg/dL was identified in 19.1% of the ASCVD population and only 4.1% had an LDL-C < 50 mg/dL. LDL control was worse in women compared to men (13.1% vs. 25.7%; P < 0.01). The average cost in one year was 3,591 American dollars (USD) per patient in primary prevention compared to 8,210 dollars per year for patients in secondary prevention (P < 0.01). While outpatient costs accounted for 59.8% of the total cost in the primary prevention group, the main cost of the secondary prevention population was related to hospital costs (54.1%).

**Conclusion:**

Despite the favorable evidence for intensive cholesterol reduction, the evaluation of large real-world database with more than 17,000 individuals showed that the targets of guideline recommendations have not yet been adequately incorporated into clinical practice. Average annual cost per patient in secondary prevention is more than twice compared to primary prevention. Hospital expenses account for most of the cost in the secondary prevention group, while outpatient costs predominate in primary prevention.

## Introduction

Atherosclerotic cardiovascular disease (ASCVD) is the leading cause of death worldwide (1,2,3,4). Brazil and other developing countries also have ASCVD as the leading cause of death and this burden is still growing in these regions (1,2,3,4).

Despite the high morbidity and mortality of ASCVD, several strategies have shown relevant reduction in the risk of complications in secondary prevention (5,6,7,8). Among these evidence-based strategies, patients with ASCVD benefit from high intensity statins and other lipid-lowering drugs to achieve progressively lower targets of LDL-cholesterol (LDL-C) (5,6,7,8). However, the application of these therapies in clinical practice has been insufficient, especially in developing countries (9,10,11,12). In details the costs of healthcare in this population (10).

Thus, a retrospective study in a national health insurance was performed to document current practice in reducing cardiovascular risk of patients with atherosclerotic disease in Brazil, and report the healthcare costs in one year of this population.

## Methods

The current project is an observational retrospective cohort study that included all patients with assessment of LDL-C in a Brazilian private health insurance between 2015 to 2017. The main aim of this study is to evaluate the levels of LDL-cholesterol and the costs of medical care in a large sample of Brazilian patients with a private health insurance. Primary objective was to assess the proportion of patients with ASCVD who had LDL-C in compliance to target levels. Secondary objective was to assess the healthcare costs in one year for patients with ASCVD (secondary prevention) or without ASCVD (primary prevention), and the distribution of LDL-C among patients without ASCVD (primary prevention).

The registry was a study with financial support from Novartis and coordinated by Instituto Americas.

### Data source

**Figure 1:**
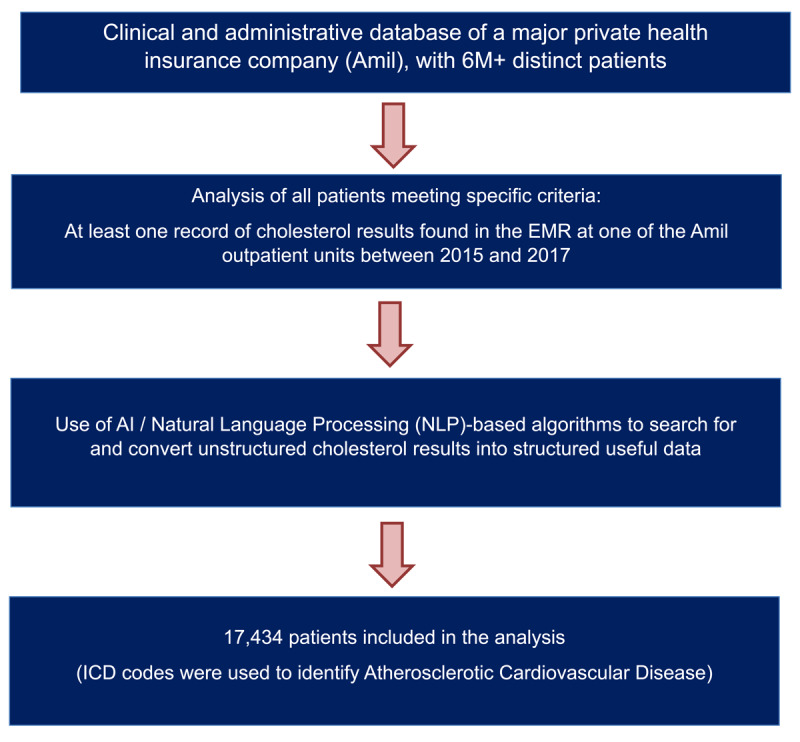
Study flowchart.

Database from a Brazilian health insurance (Amil), included around 6 million of patients followed from 2015 to 2017. The choices of the database and the period of analysis were based on the size of the population and the availability of data for cost analysis before pandemic. Since the information of LDL-Cholesterol was not structured, we used Artificial Intelligence (Natural Language Processing) algorithms to search for and convert unstructured cholesterol results into structured useful data (Figure 1).

The Natural Language Processing (NLP) solution applied in this study utilized a hybrid suite of symbolic, statistical and machine learning NLP components driven in part by a collection of clinical ontologies. At its core, the solution identifies and extracts clinical indicators such as signs, symptoms, diagnoses, labs, drugs and vitals from the narrative text of clinical documentation. Indicator attributes such as certainty, severity and date, along with contexts such as document type, document section, and whether current, historical clinical facts were also extracted. The indicators and their attributes and contexts, comprising the primitive semantic building blocks for any clinical application, are structured as discrete data elements and further refined and interpreted for clinical significance by application pertinent rules, algorithms and machine learning based analytic systems. Implemented by NLP specialists and software engineers, the clinical logic underlying the solution, as well as the content and structure of the clinical ontologies, has been designed, applied and evaluated by clinical specialists in each domain of application.

### Study population

All patients with at least 18 years old and assessment of LDL-Cholesterol were eligible. The groups were separated in primary or secondary prevention according to ICD-codes of ASCVD (Table S1). Primary prevention was considered for patients without the ICD-codes of ASCVD. All the ICD-codes were considered before the LDL-Cholesterol assessment. For this analysis it was included only patients with ICD-codes of coronary artery disease (CAD) and cerebrovascular disease since these as the most relevant atherosclerotic conditions and ICD for atherosclerotic disease in other sites could bring more heterogeneity.

### Study procedures and variables collected

The following information were assessed at baseline: age, sex, comorbidities (based on ICD-codes). Routine laboratory tests were also assessed and included not only LDL-C but also creatinine, HDL-cholesterol, triglycerides, and fasting glucose. All available patients with LDL-C were assessed based on outpatient and inpatient procedures and costs related. If more than one LDL-C exam is available during the follow-up of the same patient, the first value will be the one considered for the analysis of this study.

The study was approved by institutional review board in accordance with local regulations and was carried out in accordance with the ethical principles consistent with the Declaration of Helsinki.

### Study endpoints

The primary endpoint is the proportion of patients with ASCVD who had LDL-C < 70 mg/dL based on the guideline recommendations during the years of analysis (13,14).

The secondary endpoints included the analysis of costs in one year from the perspective of the health insurance for both groups: primary and secondary prevention. For this analysis, the dollar exchange rate was USD 1.00 = R$ 3.00, according to the average rate during the analysis period. All the health insurance claims were considered and then categorized as outpatient and inpatient costs. Costs of procedures, costs related to cardiovascular care and cost allocation were assessed. Since it was not possible to categorize the cardiovascular risks of patients in primary prevention, LDL-C < 100 mg/dL was considered as the main target, assuming a medium risk, for the average of this population (13,14,15,16,17).

### Statistical analysis

Continuous variables were described as mean (and standard deviation) or median (and interquartile range), while categorical variables as percentages with a 95% confidence interval. Categorical variables were compared by Chi-square test and continuous variables were compared by parametric or non-parametric methods, as appropriate. The primary analysis was based on the data collected and available (complete) data. Statistical analyzes were performed in R statistical software, version 3.5.2 (R Foundation for Statistical Computing).

## Results

Between 2015 and 2017, 17,434 subjects had at least one assessment of LDL-Cholesterol (Figure 1). According to ICD-10, 5,208 patients had ASVCD (29.8%; 95% CI 29.1%–30.5%) while the remaining were considered as primary prevention (Table 1).

**Table 1:** Baseline characteristics.


	PRIMARY PREVENTION (n = 12,226)	SECONDARY PREVENTION (n = 5,208)	TOTAL (n = 17,434)	P-VALUE

Age	54.4 (±15.9)	68.9 (±12.3)	58.8 (±16.3)	<0.01

Female Sex	69.4%	52.2%	64.2%	<0.01

Hypertension	52.2%	79.6%	60.4%	<0.01

Diabetes Mellitus	33.7%	56.2%	40.4%	<0.01

Dyslipidemia	32.2%	43.6%	35.6%	<0.01

CKD	4.6%	18.2%	8.7%	<0.01

LDL-cholesterol	114.3 (±35.1)	119.6 (±36.9)	115.9 (±36.2)	<0.01

HDL-cholesterol	48.2 (±16.2)	42.7 (±12.5)	46.6 (±15.4)	<0.01

Triglycerides	133.3 (±74.9)	144.6 (±170.5)	136.7 (±140.4)	<0.01

HbA1C	6.7 (±3.7)	6.6 (±3.6)	6.6 (±3.6)	0.10


The values are presented as mean (and standard deviation) and percentages. ^†^ CKD means chronic kidney disease.

### Baseline characteristics

The overall mean age was 58.8 (±16.3) years with an older population in the secondary prevention group (Table 1). Most patients were female in both groups with a higher percentage of them in the primary prevention population (69.4% vs. 52.2%; P < 0.01). Comorbidities as hypertension, dyslipidemia, diabetes mellitus and renal chronic disease were more common in the population with ASCVD.

### LDL cholesterol in the population with ASCVD

In the secondary prevention population (ASCVD), 19.1% of the patients had a LDL-C < 70 mg/dL (Table 2) and the percentage of patients on the targets of guideline recommendations was worse in women compared to men (13.1% vs. 25.7%; P < 0.01). Using a stricter target (<50 mg/dL), 4.1% were in this more controlled group (Table 2).

**Table 2:** Distribution of LDL cholesterol values in the population with and without ASCVD.


LDL-CHOLESTEROL	PRIMARY PREVENTION (n = 12.226)	SECONDARY PREVENTION (n = 5.208)	TOTAL (n = 17.434)

< 50 mg/dL	1.7%#	4.1%#	2.4%

50 - 69 mg/dL	6.8%#	15.0%*	9.2%

70 – 99 mg/dL	26.2%#	31.7%	27.8%

100 – 129 mg/dL	33.4%*	26.9%	31.4%

130 – 159 mg/dL	20.4%	14.4%	18.6%

160 – 189 mg/dL	8.2%	5.0%	7.3%

≥ 190 mg/dL	***3.5%***	***2.8%***	3.3%


On-target# Boderline* Out of the target ***Suspicion of Familial Hypercholesterolemia***

**Table 3:** Total cost per patient per year in primary and secondary prevention (outpatient and inpatient).


METRIC	w/o ASCVD	w/ ASCVD	TOTAL

**Total cost per patient per year**	**$3,591**	**$8,210**	**$4,971**

**Outpatient cost per patient per year**	**$2,118**	**$3,575**	**$2,554**

ED cost per patient per year	$171	$241	$192

OP Exam/Procedure/Therapy cost per patient per year	$1,378	$2,451	$1,698

Elective office visit cost per patient per year	$570	$883	$663

OP Non-invasive imaging tests cost per patient per year*	$591	$1314	$807

**Inpatient cost per patient per year**	**$1,473**	**$4,635**	**$2,417**

Medical (non-surgical) IP cost per patient per year	$618	$2,921	$1,306

Cardiovascular surgical cost per patient per year*	$165	$2,331	$812


ED means emergency department; OP means outpatient; High complexity includes non-invasive imaging tests.

### LDL cholesterol in the population without ASCVD

In the primary prevention population (without ASCVD), 65.3% (CI 95% 64.4–66.1%) had an LDL-C > 100 mg/dL with 3.5% above 190 mg/dL (Table 2).

### Healthcare costs

**Figure 2:**
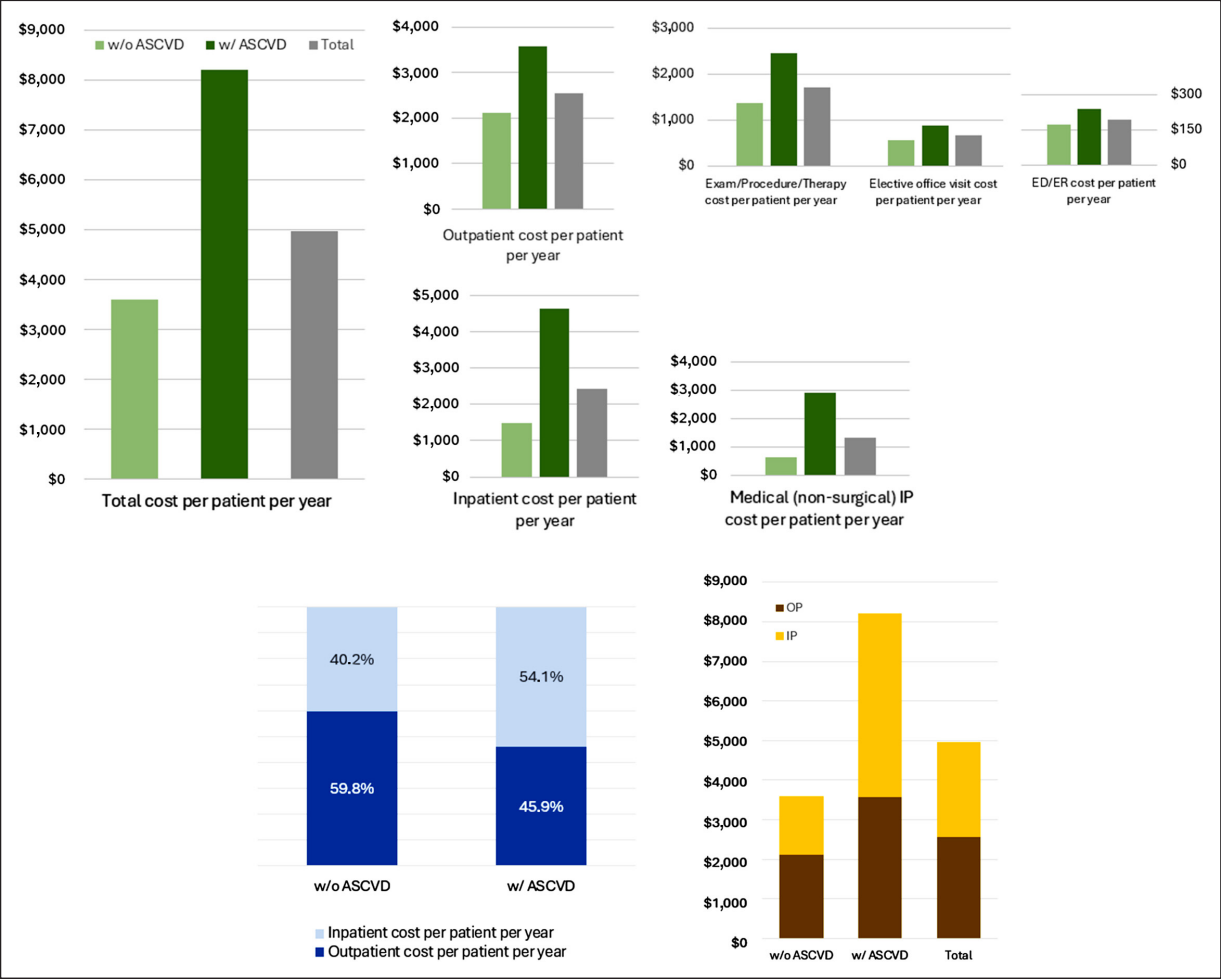
Average costs in primary and secondary prevention (outpatient and inpatient).

The average cost in one year was USD 3,591 per patient in primary prevention compared to a mean cost of USD 8,210 per year per patient in secondary prevention (P < 0.01). While outpatient costs accounted for 59.8% of the total cost in the primary prevention group, in the secondary prevention population, most of the total cost was related to hospital costs (54.1%). (Table 3, Figure 2 and Table S2) and to cardiovascular care (Table S3). Among patients with ASCVD, most of the costs were related to medical fees (Table S4).

## Discussion

The current real-world data study assessing more than 17,000 subjects followed in a private health insurance in Brazil, identified almost 30% of the population with ASCVD. Most patients were female, and, among all risk factors, hypertension was the most common (present in almost 80% of patients in secondary prevention). LDL-Cholesterol was above a minimum target of 70 mg/dL in approximately 80% of the population with ASCVD which represents an important need not attended yet in clinical practice.

Intensive lipid control is a fundamental part of the strategy to reduce the cardiovascular risk of patients with ASCVD (13,14,15,16,17). Global data have shown a reduction in LDL-Cholesterol along the years but still with a large percentage of patients with levels above the guideline recommended goals (18). Multinational studies performed in European countries revealed that only approximately 30% of very-high risk patients reached the LDL-C target of <70 mg/dL (19,20). A similar finding was obtained in the American GOULD registry (21). The lower percentage of patients with LDL-C < 70 mg/dL in the current analysis may reflect not only regional variations but mainly the difficulty in reducing LDL-Cholesterol, especially with the medications available at the time of the analysis (18,22,23). Considering the years from 2015 to 2017, the absence of formal recommendation of adjunctive therapies like anti-PCSK9 may justify part of the patients outside the target. Nevertheless, more recent registries have shown that even with lower targets and the formal recommendation of adding ezetimibe and/or anti-PCSK9, still more than half of the patients do not reach guideline recommended goals (18,19,20,21,22). Understanding the limitations of these patients to achieve the target of LDL-Cholesterol is a critical step to reduce the risk of this population.

In the primary prevention group, the guideline recommended LDL-C < 100 mg/dL was present in only 34.7%. This group represent most of the global population and reducing LDL-Cholesterol in this predominant group would not only impact a numerically higher number of subjects but would reduce the risk of a first cardiovascular event and, as consequence, reduce the number of patients with ASCVD (24). Beyond the importance of reducing the LDL-Cholesterol levels in this population, 3.5% of this group had a LDL-C ≥ 190 mg/dL which is indicative of higher suspicion to familial hypercholesterolemia (25,26,27). The current percentage is higher than previous literature which also showed low investigation among these patients with LDL-C ≥ 190 mg/dL (27). Despite variations in the threshold for investigation (25,26,27), the assessment of the diagnosis of familial hypercholesterolemia should be considered not only to evaluate more precise therapies to the patients but, especially, to support decision of early screening in the family of the patient.

Finally, regarding the healthcare costs, the average cost per patient in secondary prevention is more than twice the annual cost in primary prevention. Hospital costs account for most of the cost in the secondary prevention group, while outpatient costs predominate in primary prevention. In an era of value-based healthcare (28,29), such information will be useful in planning clinical care pathways and health policies. Beyond the clinical benefits measured in clinical trials that support guideline recommendations, such as lipid control in primary prevention, information regarding costs is essential to define strategies to avoid the development of ASCVD also in terms of health economy (28,29).

The current analysis had some limitations. Only a small part of the health insurance population had LDL-cholesterol available. This could be a consequence of healthy subjects covered by the insurance that do not perform regular evaluation of lipide profile and because the artificial intelligence (AI) algorithm could not capture all the data. Information regarding therapies to reduce cholesterol in the overall population also. Other limitation was the assumption of a target of LDL-C < 100mg/dL in primary prevention population but cardiovascular risk categories of the primary prevention group were not available. Other aspects that deserve to be mentioned include the fact that the diagnosis was based on ICD-codes, did not include peripheral arterial disease and aortic disease, and the assessment of laboratory results was based on routine and not systematic exams. Nevertheless, the current analysis represented the real medical practice about the main causes of atherosclerotic syndromes and was possible to analyze not only the levels of LDL-Cholesterol but the costs of medical care in a large sample of Brazilian patients with a private health insurance, which were the main objectives of the study.

## Conclusion

In a multicenter real-world database with more than 17,000 individuals it was shown that the goals of LDL-cholesterol lowering have not yet been adequately incorporated into clinical practice with a worse control in women. Average annual cost per patient in secondary prevention is more than twice compared to primary prevention. Hospital costs account for most of the cost in the secondary prevention group, while outpatient costs predominate in primary prevention. Finally, this information is a call for action to improve lipid control in Brazil and could be useful in planning clinical care pathways and health policies aimed at efficiency and delivery of value-based healthcare.

## Data Accessibility Statement

Anonymized participant data can be made available upon requests directed to the corresponding author. Proposals will be reviewed based on scientific merit. After approval of a proposal, data can be shared through a secure online platform after signing a data access agreement.

## Additional File

The additional file for this article can be found as follows:

10.5334/gh.1345.s1 Supplementary Appendix. Tables S1 to S3.
