# Supplementary material for: Lipid Control and Medical Costs Among Patients With and Without Established Atherosclerotic Cardiovascular Disease Followed in a Brazilian Private Healthcare System
Source: Glob Heart. 2024 Aug 14;19(1):65. doi: 10.5334/gh.1345 (PMC11328683; doi:10.5334/gh.1345)
Supplement: Supplementary Appendix. — Tables S1 to S3. [file gh-19-1-1345-s1.pdf]

Supplementary Appendix

ICD codes 2
Table S1. Cost per procedure in US dollars 3
Table S2. Total cost per patient per year in primary and secondary prevention (outpatient and inpatient) 4
Table S3. Cardiovascular and Non-cardiovascular costs 5
Table S4. Cost allocation 6

ICD codes

Atherosclerotic Cardiovascular Disease (ASCVD): The following ICD-codes were considered for ASCVD group: I20, I21, I22, I23, I24, I25, I63, I64, I65, I66, I67, I68, I69 and I70.

Table S1. Cost per procedure in US dollars

Main procedure	Unitary cost
Aneurysm procedure	$15,678
Congenital cardiac disease	$10,736
PCI	$4,020
Valve surgery	$20,647
Pacemaker	$8,394
CABG	$18,527
Others	$3,772

PCI, percutaneous coronary intervention; CABG, coronary artery bypass surgery

Table S2. Total cost per patient per year in primary and secondary prevention (outpatient and inpatient)

Metric	w/o ASCVD	w/ ASCVD	Total
**Total cost per patient per year**	**$ 3,591**	**$ 8,210**	**$ 4,971**
**Outpatient cost per patient per year**	**$ 2,118**	**$ 3,575**	**$ 2,554**
ED cost per patient per year	$171	$241	$192
OP Exam/Procedure/Therapy cost per patient per year	$ 1,378	$ 2,451	$ 1,698
Elective office visit cost per patient per year	$ 570	$ 883	$ 663
OP Non-invasive imaging tests cost per patient per year*	$ 591	$ 1314	$ 807
**Inpatient cost per patient per year**	**$ 1,473**	**$ 4,635**	**$ 2,417**
Medical (non-surgical) IP cost per patient per year	$618	$ 2,921	$ 1,306
Cardiovascular surgical cost per patient per year*	$165	$ 2,331	$ 812

ED means emergency department; OP means outpatient; High complexity includes non-invasive imaging tests.

Table S3. Cardiovascular and Non-cardiovascular costs

Values	s/ ASCVD	c/ ASCVD
IP Cardio ($ per mbr)	$165	$2,331
IP NoCardio ($ per mbr)	$1,309	$2,307
IP Cardio (events per mbr)	0,038	0,358
IP NoCardio (events per mbr)	0,369	0,563
IP Cardio (Unit Cost)	$4,332	$6,517
IP NoCardio (Unit Cost)	$3,544	$4,097
OP High Cmplexity ($ per member)	$591	$1,314
OP Low Cmplexity ($ per member)	$370	$471

IP=inpatient; OP=outpatient

IP Cardio ($ per mbr) = total cost of hospitalizations for cardiovascular causes divided by the number of patients in the group (with or without ASCVD)

IP NoCardio ($ per mbr) = total cost of hospitalizations for non-cardiovascular causes divided by the number of patients in the group (with or without ASCVD)

IP Cardio (events per mbr) = number of hospitalizations for cardiovascular causes divided by the number of patients in the group (with or without ASCVD)

IP NoCardio (events per mbr) = number of hospitalizations for non-cardiovascular causes divided by the number of patients in the group (with or without ASCVD)

IP Cardio (Unit Cost) = average cost of hospitalization due to cardiovascular causes

IP NoCardio (Unit Cost) = average cost of hospitalization for non-cardiovascular causes

OP High Complexity ($ per member) = total cost of highly complex outpatient exams and procedures divided by the number of patients in the group (with or without ASCVD)

OP Low Complexity ($ per member) = total cost of low complexity outpatient exams and procedures divided by the number of patients in the group (with or without ASCVD)

Table S4. Cost allocation

IP cost groups	IP_NaoCardio	IP_Cardio
OPSM	31.4%	4.,9%
Medical fees	21.9%	30.3%
Standard Material	16.3%	9.0%
Standard Medication	12.4%	5.4%
Hospital daily rates	6.5%	5.6%
Taxes	4.7%	2.1%
Therapies	2.6%	1.4%
Others	4.2%	4.2%

OPSM, orthotics, prosthetics and special materials
